# A Case of Lithium Encephalopathy with Therapeutic Lithium Levels: The Diagnostic Role of EEG

**DOI:** 10.1155/2022/8052471

**Published:** 2022-12-16

**Authors:** Claudia Carmassi, Benedetta Nardi, Simone Battaglini, Chiara Bonelli, Miriam Violi, Enrica Bonanni, Liliana Dell'Osso

**Affiliations:** ^1^Department of Clinical and Experimental Medicine, University of Pisa, Pisa, Italy; ^2^Department of Neurosciences, Neurology Unit, University of Pisa, Pisa, Italy

## Abstract

*Introduction*. Lithium is considered a first-line therapy for both the acute phase and the maintenance of bipolar disorder. Many studies highlighted its neuroprotective and neuroplastic capacity suggesting a potential usefulness in the treatment of neurodegenerative diseases. Despite the undeniable efficacy, lithium clearly presents several adverse effects including neurotoxicity, also known as lithium encephalopathy, regarding both neurological, psychiatric, and cognitive side effects. In this case, adverse reactions are not always related to its serum levels, possibly appearing within the therapeutic range. *Case Presentation*. We analyzed the case of a bipolar patient who has been uncontinuosly treated with lithium salts since the onset of the psychopathological picture. Over the years, the average values of lithemia always remained around 0.60-0.70 mEq/L, but in 2019, the patient begun to manifest distal tremors and in the mandibular district accompanied, in the following months, by psychomotor slowdown, generalized tremors, reduced alertness, spatiotemporal disorientation, and aphasia. While alterations referable to neurodegenerative diseases were excluded, EEG maintained rhythm alteration 1 year after the probable intoxication. *Discussion*. This case confirms the central role of EEG for lithium neurotoxicity, while its dosages are in therapeutic range, being plasma levels are not always indicative of liquoral and neuronal lithium's levels. We highlight the importance of an early diagnosis of lithium encephalopathy proposing EEG as an indispensable tool for assessing lithium neurotoxicity both in acute and chronic intoxication.

## 1. Introduction

Since the discovery of its medical properties, lithium has been one of the main protagonists of the pharmacological treatment of many psychiatric disorders [1–3]; its popularity certainly became unmatched in the late ‘40s with its application in the therapy of affective disorders [4, 5]. Despite the discovery and introduction of a new generation of mood stabilizers, lithium is still considered a first-line therapy for both the acute phase and maintenance of bipolar disorder (BD) [6, 7] and an additional treatment in major depression [8, 9]. To his fame and longevity, surely contributed the fact that to this day, it remains the only compound with established antisuicidal properties [10–14] and the only whose prophylactic effect is proven on both new depressive and maniacal episodes [3, 15, 16].

Lithium's mechanisms of action underlying its mood-stabilizing properties have not yet been fully clarified [17]. Many studies highlighted its effect on preventing neural apoptosis and increasing the excretion of neurotrophic factors [18], modulating autophagy, oxidative stress and overregulation of mitochondrial function [19], and reducing the proinflammatory state [20], thus explaining its suggested neuroprotective and neuroplastic capacity [8, 21, 22]. Recent findings also suggest its usefulness in the treatment of neurodegenerative diseases, neurodevelopmental disorders, and hypoxic ischemic/traumatic brain injury [8]. A recent study, interestingly, investigated the presence of biomarkers that could predict acute and long-term treatment response to a lithium therapy in BD patients [23]. Despite the undeniable efficacy and the presence of a retard formulation with greater efficacy and lower adverse effects [24], lithium clearly presents some management issues due to its narrow therapeutic index and several potential side effects that could limit its use in clinical practice [25, 26].

Particularly, a prolonged administration can lead to damage of the kidney's primary via tubular damage—resulting in polyuria and neurogenic diabetes insipidus—and glomerular damage [27] and alterations in thyroid's function, mainly hypothyroidism and more rarely hyperthyroidism [28, 29]; for these reasons, regular monitoring of renal and thyroid function is highly recommended during treatment [26]. Moreover, lithium can lead to increased serum levels of parathyroid hormone and calcium, in some cases, resulting in hyperparathyroidism and hypercalcemia [30] and in a possible aggravation of some dermatological pathologies such as psoriasis [26, 31].

Another highly relevant concern is its referred neurotoxicity, also known as lithium encephalopathy, regarding both neurological, psychiatric, and cognitive side effects. In this case, adverse reactions are not always related to its serum levels, possibly appearing within the therapeutic range, for plasma levels may not always be indicative of liquoral and neuronal lithium's levels [32], and may be both reversible or irreversible depending on the timeliness of identification and treatment [33].

Neurotoxicity can occur during the first days of treatment as well as after years of maintenance therapy and is characterized by neurological signs (ataxia, dysarthria, tremor, reduced motor coordination, speech articulation alteration, rigidity, extrapyramidal symptoms, asthenia, fasciculations, and convulsions), cognitive signs (temporal-spatial disorientation, concentration, comprehension, mnemonic, and associative ability deficits), and psychic signs (restlessness, irritability, confusion, bizarre behavior, and delirium) [32, 34].

In this paper, we aim to describe a peculiar case on lithium-induced neurotoxicity happened within the therapeutic range of lithemia, after many years of treatment.

## 2. Case Presentation

Q. X. is a 63-year-old woman with a long history of BD with mixed episodes. She is unmarried, retired, and lives alone. Q. X. shared a positive anamnesis for mood disorders with her mother, who suffered from a depressive-anxious disorder and her niece who was recently diagnosticated with BD type I; none of them have been treated with lithium. Since adolescence, she showed cyclothymic-irritable temperament with an inclination to impulsivity, affective instability, and sudden anger's outbursts that caused impairment of interpersonal relationships. The onset of the psychopathological picture goes back to the age of 19 when, following her sister's sudden death, she experienced depressive mood with dysphoric cues, apathy, abulia, and elevation of anxiety, Hyperarousal, intrusive thoughts, and reexperiencing. The symptomatology resolved spontaneously without needing a pharmacological treatment. After 11 years, consequently to the ending of a sentimental relationship, she underwent a resurgence of the previously described picture; this time, addressing a psychiatrist who prescribed a therapy based on lithium salts, at unspecified dosage. In the years, she was followed by many specialists who discontinued treatment with lithium salts and carried out several unspecified therapeutic modifications, with little psychoaffective compensation, only to reintroduce lithium in 2008 (lithium carbonate up to 750 mg/day), over time variously associated with antipsychotics, antidepressants, and benzodiazepines, with a good psychoaffective and sociooccupational compensation. Over the following years, the average values of lithemia always remained around 0.60-0.70 mEq/L. In 2019, at 61, the patient begun to manifest distal tremors in the extremities of the upper limbs and in the mandibular district. For this reason, in January 2020, she underwent a cerebral SPECT with DaTscan whose results were not compatible with a nigrostriatal degeneration.

In September 2021, following her retirement, she faced a resurgence of the symptomatology with depressed mood, reduced energy level, irritability, social withdrawal, increased anxiety levels, and feelings of emptiness and loneliness with a tendency to clinophilia, referring also an aggravation of the tremors for which she received a diagnosis of “Essential Tremor” and a prescription for propanolol without however perceiving a clinical benefit and therefore, subsequently and independently suspended. In December 2021, for the worsening of the tremors that now also involved the lower limbs and three episodes of sudden fall accompanied by psychomotor slowdown, generalized tremors, reduced alertness, spatiotemporal disorientation, and aphasia, Q.X. made numerous accesses to the local E.R. without however referring any clinical improvement. She was therefore admitted to the local hospital psychiatric department where she underwent a dosage of lithemia (0.83 mEq/L at the time of hospitalization) and the complete interruption of the psychopharmacological therapy. Five days later, Q.X. was transferred to the neurologic department where she underwent a rachicentesis, resulting negative for autoimmune encephalopathy autoantibodies, TAU/B-amyloid protein and protein 14.3.3, a brainstem MRI that did not show any significant alteration, and CT scan that excluded the presence of blood collections and focal densitometric alterations. The neurological evaluation deposed for a case of extrapyramidal disease a treatment with levodopa/carbidopa up to 100/25 mg/day was suggested, still without benefits. During the hospitalization, Q.X. showed a progressive improvement in vigilance, spatiotemporal orientation, and motor skills.

One month later, Q.X. was discharged with a final diagnosis of “Acute behavioral decompensation in subjects with BD with negative neurological investigations” and a therapy based on valproic acid (750 mg/day), quetiapine (100 mg/day), and diazepam (8 mg/day). During the following days, Q.X. re-presented a depressive mood, elevated anxiety and internal tension, emotional lability with crying crisis, social withdrawal, and tendency to clinophilia. For this reason, she was then hospitalized in the psychiatric ward of Pisa Hospital. At the beginning of hospitalization, Q.X. showed distal tremors in the upper limbs bilaterally and at the mandibular region. She underwent a routine ECG and blood test resulting normal except for a slight anemia and minor alteration in the metabolic profile. Electrolytes, coagulation profile, renal, and thyroid function were found normal. An EEG was performed (Figures 1 and 2), reporting a symmetrical trace, consisting of a background rhythm at 8-9 Hz, not very regular, distributed on the posterior regions, reacting to the opening of the eyes, interspersed at rapid bianterior rhythms, and isolated potential theta sometimes of pointed appearance mainly anterior and resulting in “isolated pointed anomalies with anterior prevalence” and a neurological consultation suggested a possible compatibility with lithium encephalopathy. During the hospital stay, Q.X. was treated with valproic acid (900 mg/day), quetiapine (100 mg/day), paroxetine (20 mg/day), trimipramine (35 mg/day), diazepam (7 mg/day), and pramipexole (0.36 mg/day) showing a progressive improvement with the reduction of the tremor until complete disappearance, mood improvement, reduction of the internal tension and anxious quota, disappearance of emotional lability, and adequate thinking in form and content. The improvement was such as to allow her discharge from the hospital.

## 3. Discussion

We analyzed the case of a bipolar patient who experienced a lithium neurotoxicity despite a normal lithemia.

For the past eighty years, lithium has been the preferred therapy for the long-term management of bipolar patients [35–37] leading to a better outcome in both BD-I and BD-II patients [38]. The neurotoxic effect of elevated lithium plasma levels is well known and documented [32, 39]; however, literature reporting cases of encephalopathy occurring while in the therapeutic range is still scarce [33, 39].

To the best of our knowledge, the first occurrence was described by West et al., who reported cases of neurotoxicity in patients in manic or psychotic phase with a psychopharmacologic therapy based on lithium only, in therapeutic range [39]. Later, Fetzer et al. [40] collected three cases of patients with organic brain syndrome and neuropsychological dysfunction, whose EEG showed abnormal slow-diffused theta activity, treated with lithium in addition to neuroleptic and with lithium level's range between 0.7 mEq/liter and 1.3 mEq/liter. The encephalopathy was resolved only interrupting the therapy and led Fetzer to hypothesize that a conjunction between lithium at therapeutic doses and neuroleptics could cause neurotoxicity [40].

More recently, Sarappa et al. described a case of possible lithium encephalopaty referring to EEG as important instrument for early reporting of lithium neurotoxicity when dosages are in therapeutic range [33]. Although Q.X. arrived late at our observation and the symptoms of a possible encephalopathy did not express an acute intoxication, we found similarities with Sarappa et al.'s case.

In both cases, the scintigraphy excluded alterations referable to neurodegenerative diseases and comparing the EEGs, a common background trace characterized by diffuse alteration with unstable dominant activity at 8-8.5 cycles/sec mixed with theta waves was highlighted [33]; moreover, the symptoms appeared after a progressive suspension of the lithium. Interestingly, Q.X. maintained an EEG rhythm alteration 1 year after the probable intoxication, confirming the central role of EEG in detecting chronic lithium neurotoxicity.

Ultimately, lithium effective therapy in BD has a limited tolerability even when its dosages fall within the therapeutic range [33, 40].

The case of Q.X. highlights the importance of an early diagnosis of lithium encephalopathy and, in accordance with previous literacy, proposes EEG as an indispensable tool for assessing lithium neurotoxicity both in acute and chronic intoxication. In our case, we decided to not reintroduce lithium; instead, we initiated a mood-stabilizing therapy with valproate, after evaluating hepatic functionality and the risk/benefit ratio. Previous studies suggested a possible reintroduction of lithium at the lowest dose after the complete remission of the symptoms, but also reported, soon after the reintroduction, the reactivation of neurological adverse effects [41].

Finally, a note of interest should also be given to the patient's psychiatric history which represents an excellent example of how the trajectory of the disease may evolve during the course of life. On an underlayer of vulnerability, portrayed by autistic traits and personality traits belonging to cluster B, a major trauma represented by the sudden loss of a close relative led to a posttraumatic symptomatology and to an exacerbation of a severe BD, with many recurrent mixed episodes [42–44].

## Data Availability

All data generated or analyzed during this study are included in this published article.

## Supplementary Materials

Supplementary Materials The supplementary file “EEG” reports the electroencephalogram performed on the patient during the hospitalization, also reported in Figures 1 and 2. A written consent for publication was given by the patient, after seeing the copy of the manuscript, agreeing that the authors have removed as much identifying information as possible.
