# Supplementary material for: A Case of Lithium Encephalopathy with Therapeutic Lithium Levels: The Diagnostic Role of EEG
Source: Case Rep Psychiatry. 2022 Dec 16;2022:8052471. doi: 10.1155/2022/8052471 (PMC9788879; doi:10.1155/2022/8052471)
Supplement: Supplementary Materials — The supplementary file “EEG” reports the electroencephalogram performed on the patient during the hospitalization, also reported in Figures 1 and 2. A written consent for publication was given by the patient, after seeing the copy of the manuscript, agreeing that the authors have removed as much identifying information as possible. [file 8052471.f1.zip › 8052471.f1/Consent_to_publication_form-2.pdf]

Consent to publication

Hindawi

I agree that information about me and/or my photographs can be published in a Hindawi journal (https://www.hindawi.com/journals/). I understand that:

- Although my name will not be published and the authors will remove as much identifying information as possible, complete anonymity is not guaranteed and I might be identified from the published article.
- Hindawi articles are published online under a Creative Commons Attribution License (https://creativecommons.org/licenses/by/4.0/) that allows anyone to reuse the article and its images provided they give credit to the authors.
- The manuscript may change during the review and production processes.
- Once the article is published, I cannot remove my consent.

Name of subject described and/or pictured:
Stefania Papucci

If the subject is a minor (child), unable to consent, not competent, or deceased, I agree for them. Name of person giving consent (if different):

Relationship to subject of person giving consent (if different):

Manuscript title:
A case of lithium encephalopathy with therapeutic lithium levels: the diagnostic role of EEG

Author:
Carmassi Claudia, Nardi Benedetta, Battaglini Simone, Bonelli Chiara, Violi Miriam, Bonanni Enrica, Dell'Osso Liliana

I have seen a copy of the manuscript: X
or
I waive the right to see a copy of the manuscript: •

Signature of subject

Date: 10/09/2022

Signature of author:

Date: 10/09/2022

This form will be retained by the authors and made available to Hindawi only on request
