# Supplementary figures and images for: A Case of Lithium Encephalopathy with Therapeutic Lithium Levels: The Diagnostic Role of EEG
Source: Case Rep Psychiatry. 2022 Dec 16;2022:8052471. doi: 10.1155/2022/8052471 (PMC9788879; doi:10.1155/2022/8052471)

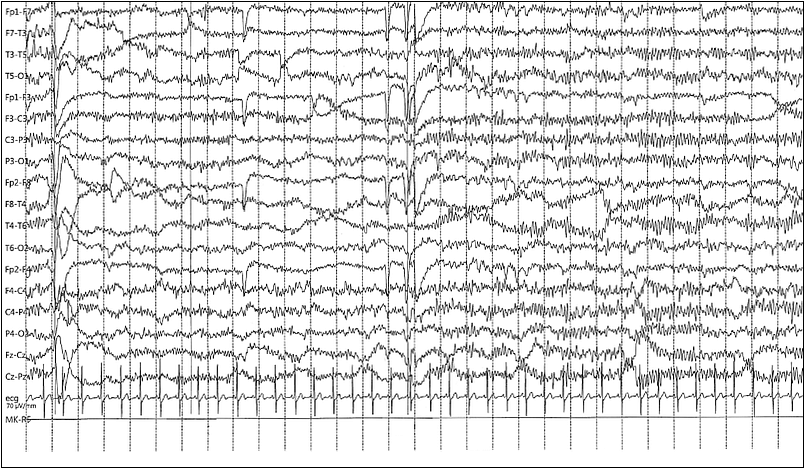


Closed eyes

Open eyes

08 35 55

08 36 25

Open mouth

Supplement: Supplementary Materials — The supplementary file “EEG” reports the electroencephalogram performed on the patient during the hospitalization, also reported in Figures 1 and 2. A written consent for publication was given by the patient, after seeing the copy of the manuscript, agreeing that the authors have removed as much identifying information as possible. [file 8052471.f1.zip › 8052471.f1/EEG_1_b (1).docx]

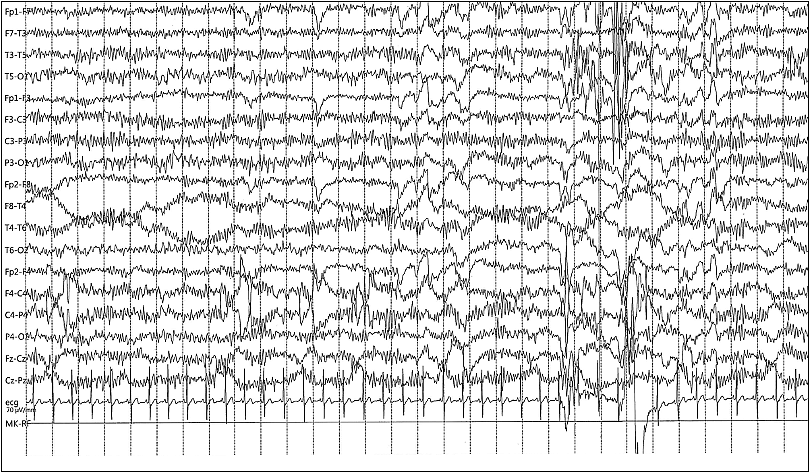


08 36 55

08 36 25

Artifact

Supplement: Supplementary Materials — The supplementary file “EEG” reports the electroencephalogram performed on the patient during the hospitalization, also reported in Figures 1 and 2. A written consent for publication was given by the patient, after seeing the copy of the manuscript, agreeing that the authors have removed as much identifying information as possible. [file 8052471.f1.zip › 8052471.f1/EEG_2_b (1).docx]
